# Calcification, Posterior Acoustic, and Blood Flow: Ultrasonic Characteristics of Triple-Negative Breast Cancer

**DOI:** 10.1155/2022/9336185

**Published:** 2022-09-26

**Authors:** Kangjian Wang, Zongkai Zou, Haolin Shen, Guimei Huang, Shuping Yang

**Affiliations:** ^1^Department of Ultrasound Medicine, Zhangzhou Affiliated Hospital of Fujian Medical University, Zhangzhou, China; ^2^Department of Pathology, Zhangzhou Affiliated Hospital of Fujian Medical University, Zhangzhou, China

## Abstract

Previous studies suggest that triple-negative breast cancer (TNBC) may have unique imaging characteristics, however, studies focused on the imaging characteristics of TNBC are still limited. The aim of the present study is to analyze the ultrasonic characteristics of TNBC and to provide more reliable information on imaging diagnosis of TNBC. This retrospective study was performed including 162 TNBC patients with 184 TNBC lesions. 174 non-TNBC cases with 196 lesions were used as the control group. The median size of TNBC lesions and non-TNBC lesions were 23 mm × 16 mm and 21 mm × 15 mm, respectively. The shape of most breast cancer lesions was irregular. However, 15.30% (28/183) TNBC lesions and 16.84% (33/196) non-TNBC lesions were oval-shaped. Most breast cancer lesions (79.78% TNBC & 85.71% non-TNBC) were ill-defined. In comparison to non-TNBC, the distinctive ultrasonic characteristics of TNBC were summarized as three features: calcifications, posterior acoustic, and blood flow. Microcalcifications was less common in non-TNBC. The remarkable posterior acoustic characteristics on TNBC were no posterior acoustic features (136, 73.91%). Avascular pattern (21.74%) was also more common in TNBC. The other feature of TNBC was markedly hypoechoic lesions (23.91%). The above-mentioned differences between TNBC and non-TNBC were significant. 93.48% TBNC and 94.39% non–TNBC lesions were in BI-RADS-US category of 4A-5. The results indicate that TNBC has some distinctive ultrasound characteristics. Ultrasound is a useful adjunct in early detection of breast cancer. A combination of ultrasound with mammography is excellent for detecting breast cancer.

## 1. Introduction

Breast cancer is the leading cancer in women [1]. About 30% of new cancer cases in women are breast cancer [1]. Fortunately, the prognosis of breast cancer has greatly improved [1]. Imaging examination plays a predominant role in screening diagnosis and monitoring of breast cancer [1–5]. According to the guidelines, mammography is considered as the first-line imaging method of breast cancer screening [2–5]. Ultrasonography (US) can also provide supplemental information about early detection of breast cancer, especially among women with dense breasts [2–6]. Women with dense breast tissues are more common in China [3, 5]. Mammography has relatively lower sensitivity in women with dense breasts [3, 5]. Ultrasound is a useful supplemental method of screening and early detection for breast cancer [6, 7].

Triple-negative breast cancer (TNBC), which negatively expresses estrogen receptors (ER), progesterone receptors (PR), and human epidermal growth factor receptor 2 (HER2), is a distinctive subcategory of breast cancer [7]. Triple-negative breast cancer accounts for about 10–15% of all breast cancers [8–11]. Xing et al. [12] have reported 29.57% of TNBC among 328 breast cancer women in Southern Shaanxi Province, China. Triple-negative breast cancer has a relatively poor prognosis due to more aggressive biological characteristics [11, 13]. In 2008, Yang et al. [14] reported different mammographic features of TNBC in young women. Thereafter, several studies analyzing the US characteristics of TNBC have been carried out in China [15–18], Japan [19], and South Korea [20, 21]. Recently, Tian et al. [22] reviewed studies based on the US features of TNBC. In the meta-analysis, only 620 patients with TNBC in ten clinical studies were included [22]. In consideration of the large number of patients with breast cancer, studies focused on the imaging characteristics of TNBC are still limited. Previous studies suggest that TNBC may have unique imaging characteristics different from other subcategories of breast cancer [22]. However, the results of previous studies are not inconsistent. Wang et al. [18] indicated that a great diversity of ultrasonic characteristics were found in the TNBC. The pathological grade, the expression level of Ki67 and the HER2 score had significant effect on the tumor shape and the presence of calcifications [17]. Some lesions of TNBC may present similar sonographic appearances to breast fibroadenomas [23]. Considering its more aggressive biological characteristics and poor outcome, imaging tools for early detection for TNBC need to be further explored. In the current study, the purpose is to analyze the US characteristics of TNBC and to provide more reliable information for imaging diagnosis of TNBC.

## 2. Method

### 2.1. Patient Selection

Using the Medical Information System, “triple-negative” was used as a diagnostic message to screen for cases with TNBC. This retrospective study included consecutive TNBC inpatients who received therapy for breast cancer at Zhangzhou Affiliated Hospital of Fujian Medical University between January 1st, 2018 and December 31, 2020. Inclusion criteria were as follows: 1. pathologically confirmed TNBC; 2. ultrasonography was performed in our hospital before TNBC was pathologically confirmed. Exclusion criteria included: 1. male; 2. missing data. Due to the fact that incidence of non-TNBC is much higher than that of TNBC [12], consecutive non-TNBC cases discharged from April 1st, 2019 to June 30, 2019 as the control group were analyzed. An ethical application was submitted to the ethics committee of Zhangzhou Affiliated Hospital of Fujian Medical University. This retrospective study has been approved by the ethics committee (2021LWB099). The current study strictly followed the declaration of Helsinki. The waiver of written informed consent from the patients was approved by the ethics committee of the hospital.

### 2.2. Ultrasound Examination of the Breast and Axillary Lymph Nodes

An ultrasound examination of the breast was performed using a 7.5–10.0 MHz or higher linear probe. A comprehensive and careful real-time scan of the breasts and axillary lymph nodes was performed by experienced radiologists. The patients were in the supine position or lateral position with lifted and extended upper arms [24].

The breast lesions were categorized according to the BI-RADS classification [24]. The method of ultrasound examination was referred to as ACR BI-RADS [24]. Subcutaneous fat was used as a reference to assess the echogenicity of the tissue [24]. Location of the lesions was indicated using clock-face location and distance from the nipple [21]. A lesion was measured in at least two dimensions [24]. Characteristics of a breast lesion included size, shape (irregular, round, and oval), margin (circumscribed, indistinct, microlobulated/lobulated, crab-like, spiculated, angular, and so on), orientation (vertical, or parallel), the lesion's effect on the surrounding tissue, posterior acoustic features (enhancement, attenuating, and none), calcifications (none, microcalcification (<0.5 mm), macrocalcification (>0.5 mm), and others), and echogenicity (hypoechogenicity, hyperechogenicity, markedly hypoechogenicity, and mix hypoechogenicity) [20, 24–26]. A color Doppler US was performed when 2D ultrasound was completed. A color Doppler US was used to detect blood vessels in the breast and the lesions. A careful real-time ultrasound scan was extended to evaluate axillary lymph nodes and adjacent tissue of the breast [20, 24]. The BI-RADS classification evaluated by mammography was also collected if available.

### 2.3. Clinical Data and Pathological Data

Clinical information included patients' name, age, Tumor-Node-Metastasis (TNM) stage. The tumor-node-metastasis system was used to describe the stages of breast cancer [27]. Pathological data included diagnosis, axillary lymph node metastasis, expression of ER, PR, or HER2, and Ki-67. Immunohistochemical staining of ER, PR, and HER2 was performed. The Ki-67 proliferation index was measured. The percentage of cancer cells stained positive was detected and recorded.

### 2.4. Data Analysis

Excel and Stata software (version 12.0) were used to analyze data. The mean ± standard deviation was used to present normally distributed continuous variables. The median and interquartile were used to present skewed distributed continuous variables. The categorical variables were shown as absolute values and proportions. The clinicopathological and imaging characteristics of TNBC and non-TNBC were compared using machine learning algorithms [28]. According to the characteristics of the variables, different statistical methods were used to compare. Student's *t*-test was used to compare normally continuous variables with homogeneity of variance. Wilcoxon rank-sum test was used to compare skewed distributed continuous variables. Fisher's exact test or chi square was used for categorical variables according to the characteristics of variables. A two-tailed *p* value <0.05 was considered as statistical significance.

## 3. Method

### 3.1. General Information

From January 1, 2018 to December 31, 2020, 276 female patients with TNBC received breast cancer therapy in our hospital. 114 cases were excluded because of missing data. Finally, data on 162 patients with 184 TNBC lesions were included in the current study. In the control group, 174 patients with 196 non-TNBC lesions were included. The flow chart was presented as Supplementary-1 (Click here). The mean age of 162 patients with TNBC was 51 (25–84) years in the current study. Compared to patients with non-TNBC, no difference was found in age (P > 0.05).

### 3.2. US Characteristics of Patients with TNBC Versus Non-TNBC

The US characteristics of patients with TNBC versus non-TNBC according to the findings from enrolled patients were summarized in Table 1.

#### 3.2.1. Size of the Lesions

The median size of TNBC lesions and non-TNBC lesions were 23 mm × 16 mm and 21 mm × 15 mm, respectively. The size of TNBC lesions was larger than that of non-TNBC lesions; however, the difference was not significant (Table 1).

#### 3.2.2. Shape and Mass Margins of the Lesions

**Figure 1:**
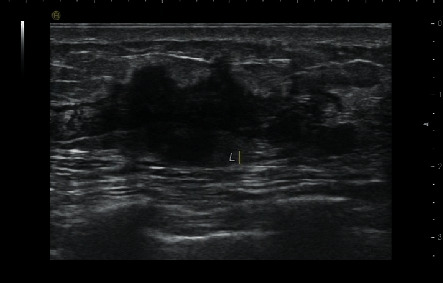
49-year-old woman with TNBC (invasive ductal breast cancer). Lesion was an irregular mass with crab-like margin and hypoechoic pattern.

**Figure 2:**
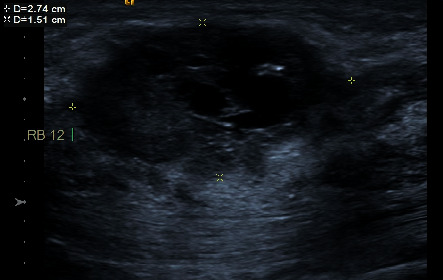
39-year-old woman with TNBC (invasive ductal breast cancer). A cystic-solid lesion with irregular mass.

The shape of most breast cancer lesions was irregular (Table 1). Nevertheless, 15.30% (28/183) TNBC lesions and 16.84% (33/196) non-TNBC lesions were oval. Viewing of the margins of lesions, most of breast cancer lesions (79.78% TNBC and 85.71% non-TNBC) were not well-defined including indistinct microlobulated/lobulated, spiculated, crab-like, or angular margins. Even so, a considerable proportion of breast cancer lesions were well-defined lesions (20.22% TNBC and 14.29% non-TNBC) (Figures 1, 2).

#### 3.2.3. Echogenicity

Most lesions with breast cancer were hypoechoic lesions (70.11% TNBC and 93.37% non-TNBC). The other echo patterns of breast cancer included remarkable hypoechoic, mix-hypoechoic, and anechoic lesions. Different echogenicity features between TNBC and non-TNBC were also shown in Table 1. Forty-four (23.91%) TNBC lesions were markedly hypoechoic lesions.

#### 3.2.4. Calcifications

The different ultrasonic characteristics of TNBC were found in calcifications and posterior acoustic features. Microcalcification was found in 64 (34.78%) TNBC lesions. Microcalcification was less common in TNBC. The difference was significant. Macrocalcification was detected in 10 (5.43%) TNBC lesions.

#### 3.2.5. The Lesion's Effect on the Surrounding Tissue

**Figure 3:**
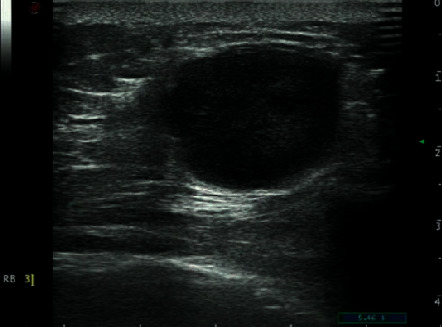
59-year-old woman with TNBC (invasive ductal breast cancer). An oral lesion with a markedly-hypoechoic pattern and microcalcification.

The lesion's effect on the surrounding tissue included architectural distortions, ductal dilatation, local edema, changes in Cooper ligaments, and so on. The above changes were not common in both TNBC and non-TNBC lesions.  Figure 3.

#### 3.2.6. Posterior Acoustic Features

**Figure 4:**
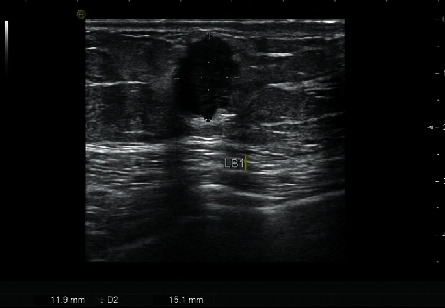
43-year-old woman with TNBC (invasive ductal breast cancer). An oral lesion with spiculated margin. Hypoechoic pattern and enhancement posterior acoustic were detected.

**Figure 5:**
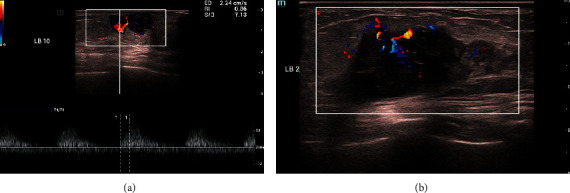
A-B 84-year-old woman with TNBC (invasive breast cancer). An irregular mass with indistinct margin and calcification. Color doppler ultrasound showed mixed and disorderly blood flow. (RI = 0.86).

The remarkable posterior acoustic characteristics on TNBC were no posterior acoustic features (136, 73.91%). Enhancement and attenuating posterior acoustic were detected in 28 and 20 lesions with TNBC, respectively. No posterior acoustic was only found in 128 (65.31%) non-TNBC lesions (Figures 4 & 5).

#### 3.2.7. Evaluation of Blood Flow by Color Doppler Ultrasound

**Figure 6:**
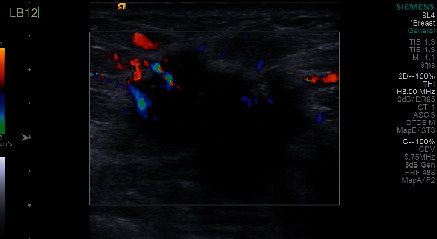
55-year-old woman with TNBC (invasive ductal breast cancer). An irregular mass with an indistinct margin and microcalcification. Color doppler ultrasound showed mixed and disorderly blood flow.

Strip or spot blood flow signals were detected in most TNBC and non-TNBC lesions; however, avascular patterns were detected in 40 (21.74%) TNBC lesions. Only two non-TNBC lesions lacked blood perfusion. The difference in blood flow between TNBC and non-TNBC was significant (*P* < 0.05). Avascular patterns were more common in TNBC lesions. Resistance indexes (RI) were recorded in 39 TNBC patients and 58 non-TNBC patients. No significant difference was found in RI between TNBC and non-TNBC patients. Thirty-five TNBC patients and 58 non-TNBC patients had RI > 0.6 (Figures 5 & 6).

#### 3.2.8. Detection of Axillary Lymph Nodes by the US

In 161 cases with TNBC (one patient with missing date about TNM), axillary lymph node metastasis was confirmed histologically in 81 patients. Among these 81 patients, 50 (61.73%) cases were preprocedure diagnosed as axillary lymphadenectasis by the US. In 101 non-TNBC patients with axillary lymph node metastasis, 68 (67.33%) cases were diagnosed as axillary lymphadenectasis by the US.

#### 3.2.9. Pathological Diagnosis and Ki-67 Index

Invasive ductal carcinoma was the most common breast cancer in both TNBC and non-TNBC. The expression of Ki-67 index was detected to be higher in TNBC lesions.

### 3.3. BI-RADS Assessed by the US and Mammography

In comparison with non-TNBC lesions, classifications BI-RADS-US category of TNBC lesions were not significantly different (Table 2). 172 (93.48%) lesions with TNBC were categorized to BI-RADS-US 4A-5, twelve lesions (6.52%) were categorized to BI-RADS 3, and 122 TNBC lesions were also examined by mammography. Among these 122 lesions, 112 (91.80%) lesions were categorized to BI-RADS 4A-5. In 196 non–TNBC lesions, 185 (94.39%) lesions were categorized to 4A-5 and eleven lesions (5.61%) were categorized to BI-RADS 3. In 155 non-TNBC lesions examined by mammography, 145 (93.55%) lesions were categorized to BI-RADS 4A-5. Ultrasound was shown as a good diagnostic method for both TNBC and non-TNBC breast cancer.

#### 3.3.1. Breast Cancer Lesions Categorized to BI-RADS 3 Assessed by the US

**Figure 7:**
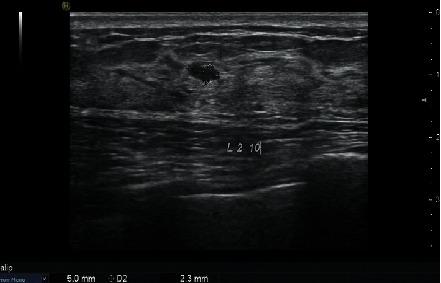
56-year-old woman with TNBC (invasive ductal breast cancer). An oral mass with a well-defined margin. The lesion with TNBC was categorized to BI-RADS three by the US.

Twelve TNBC lesions and 11 non-TNBC lesions were categorized to BI-RADS 3 by the US (Table 2). Among these 23 lesions, 16 lesions were also examined by mammography. Twelve lesions were categorized to BI-RADS 4A-5 by mammography (Figure 7).

## 4. Discussion

In the current study, the US characteristics of TNBC was summarized. Usually, breast cancer lesion was a hypoechoic mass with irregular shape and ill-defined margin. In comparison to non-TNBC, the distinctive ultrasonic characteristics of TNBC were summarized as three features: calcifications, posterior acoustic change, and blood flow. Microcalcifications were less common in non-TNBC lesions. The remarkable posterior acoustic characteristics on TNBC were no posterior acoustic features (136, 73.91%). An avascular pattern was also more common in TNBC. The other feature of TNBC was markedly hypoechoic lesions (23.91%). The above-mentioned differences between TNBC and non-TNBC were significant. Ultrasound was shown as a good diagnostic method for both TNBC and non–TNBC breast cancer. 93.48% TBNC and 94.39% non–TNBC lesions were categorized to the BI-RADS 4A-5 by the US. Ultrasound is a useful tool in the detection of breast cancer. The combination of ultrasound with mammography is excellent at screening for breast cancer.

In 2008, Yang et al. [14] first reported the mammographic features of TNBC. In this previous study, a total of 198 premenopausal women with breast cancer were included. Thirty-eight women had TNBC cancer, 67 patients had HER2+ cancer, and 93 patients had ER2+ breast cancer [14]. TNBC was more frequently associated with a mass and was less frequently associated with calcifications [14]. Irregular spiculated masses and microcalcifications, which are considered typical features of breast cancer, were less apparent in TNBC [14]. This study found remarkable mammographic differences between TNBC and HER2+/ER + cancers [16]. The authors proposed their new viewpoint that mammography may not be an ideal imaging tool for early detection of TNBC [14].

Then, a series of studies of imaging findings in TNBC were carried out [21–26, 29–33]. The previous studies focused on different samples. The mean age of TNBC patients was 46–58 years [14–17, 19–22]. In the current study, the mean age of TNBC patients was 51. Some previous studies indicated that TNBC patients were younger than non-TNBC patients [16, 25, 26]. However, no difference in age between TNBC and non-TNBC patients was found in the present study. Two studies in China also did not find that TNBC patients were younger than non-TNBC patients [15, 17]. The median size of TNBC was 23 mm × 16 mm. The size of TNBC lesions was larger than that of non-TNBC lesions, but the difference was not significant. In a recent study based on a Chinese population, the mean sizes of TNBC and non-TNBC lesions was 22 mm [17]. In Gao et al.' study [15], the mean size of 54 TNBC and non-TNBC was 23.5 mm and 23.8 mm.

Some previous studies indicated TNBC had distinctive US characteristics [14, 16, 22]. The current study showed most breast cancer lesions were hypoechoic masses with irregular shape and ill-defined margin. No differences were found in shape and margin between TNBC and non-TNBC lesions. Some previous studies also had similar results [14, 16, 17, 22]. In previous studies [21, 25], the margin of TNBC was more frequently described as lobulated or microlobulated. Another previous study [26] also indicated that TNBC was most commonly an irregular mass with ill-defined or spiculated margins. Krizmanich-Conniff et al. [26] reviewed the mass shape and mass margins of TNBC lesions described by mammography. Most commonly, the TNBC shape was irregular [26]. Most masses had ill-defined (47%), spiculated (20%), obscured (19%), or microlobulated (5%) margins [26]. Only 8% of TNBC masses had circumscribed margins [26]. Nevertheless, not all previous studies showed that TNBC presented as a typical irregular-shaped and ill-defined breast cancer mass [13, 16, 30]. In Ko et al.'s study [20], TNBC were usually irregular in shape. However TNBC were more likely to have circumscribed margins (57%). In Du et al.'s [16] study, TNBC lesions were presented as oval or round masses and 82.2% TNBC lesions had circumscribed margins. Gao et al. [15] reported that 58.1% TNBC lesions were round or oval and 37.2% TNBC lesions had circumscribed margins. However, only 22.1% non-TNBC lesion was round or oval and 3.4% TNBC lesions had circumscribed margins. An oval shape and well-circumscribed margins usually indicates a benign breast lesion [23]. In 2019, Wang et al. [31] reported that the different US features between TNBC and non-TNBC were only found in premenopausal patients but not in postmenopausal patients. TNBC in premenopausal patients was more likely to show a round or oval shape with microlobulated margins [14, 24]. In Du et al.'s [16] report, the means of age in TNBC and non-TNBC were 49 and 54, respectively. Age may be an important confounder in assessing the ultrasonic characteristics of TNBC.

Although the results of previous studies were inconsistent in the shape and margins of TNBC [14–17, 19, 20, 24–35], previous studies and the current study supported that one of distinctive US features of TNBC was less microcalcifications [12, 15, 17, 25] and less posterior echo change [16, 19, 20, 25]. Microcalcification suggests a clinical feature of ductal carcinoma in situ (DCIS) [32, 34]. In a retrospective study based on 909 cases with DCIS, microcalcifications were detected in 75% of DCIS cases [33]. Circumscribed masses without calcifications were consistent with a low incidence of DCIS in TNBC [14, 16]. A potential explanation for this phenomenon is due to rapid neoplasia lacking in precancerous stage [14, 16, 33]. Schrading et al. [35] reported that a posterior echo was observed in 67% breast cancers women with a high-risk mutation. Nevertheless, less posterior attenuating was observed in documented BRCA1 mutation and familial breast cancers [35]. A possible explanation is that the typical growth pattern of TNBC contains more internal fluid composition [36]. The nutrient vessels of TNBC may not provide enough conditions for rapid growth lesions, leading to tumor necrosis [36]. Colliquative necrosis, more often found in TNBC, results in an internal fluid component [36]. In the current study, 20% TNBC lesions lacked blood perfusion.

In Ko et al.'s [20] study, they added “markedly hypoechoic” as an echo pattern for further classification. In the study, 48% TNBC were defined as markedly hypoechoic lesions [20]. It is recommended that a markedly hypoechoic mass with a well-circumscribed margin has predictive value for the presence of TNBC [20]. Another two previous studies [21, 30] also indicate that TNBC more likely manifests as a markedly hypoechoic pattern. In the current study, a markedly hypoechoic pattern is not as common as that in previous studies. However, a markedly hypoechoic lesion is still more often observed in TNBC. A markedly hypoechoic pattern indicates an increased risk of malignancy [37]. TNBC had a higher level of Ki-67 index, which suggests highly aggressive breast cancer. Rapidly growing TNBC may outgrow their blood supply, resulting in necrosis [37]. The ultrasonic imaging findings corresponded to the pathological change [30]. A markedly hypoechoic pattern indicated the development of necrosis within TNBC tumors [30].

Cai et al. [22] analyzed previous studies which presented TNBC lacked the typical malignant US features of breast cancer. TNBC is more likely to be a regular shape without posterior attenuation and calcification. The above features make TNBC lesions similar to benign tumors [22]. However, in our study, most TNBC masses were irregular without a well-defined margin. Because of these features, 93.48% TBNC lesions were reported as BI-RADS 4A-5 by the US. The BI-RADS category scores were not significantly different between TNBC and non-TNBC lesions. Our study suggests that the US has useful diagnostic value for TNBC. Li et al. [17] reported that 104 TNBC lesions were categorized to BI-RADS 4A-6. In the lesions defined as BI-RADS 3 by the US, some of them were defined as BI-RADS 4A-5 by mammography. The combination of mammography and ultrasound is excellent at detecting breast cancer lesions.

In previous studies, heterogeneous ultrasonographic findings of TNBC were found [14–17, 19, 20, 24–27, 29–36]. Except for the above-mentioned age, other factors should be considered when ultrasonographic features of breast cancer are assessed [17]. Higher pathological grade and higher Ki67 levels were significantly associated with regular tumor shape [17]. Age, pathological grade, Ki67 level, and HER2 score may lead to heterogeneity of sonographic features in breast cancer [17].

The current study suggests that ultrasound is a good tool for breast cancer detection. The fact that some US characteristics of TNBC may affect the imaging interpretation should not be neglected. In Yeo et al.'s study [38], ultrasound elastography, and color Doppler imaging of 63 TNBC lesions were assessed by three blinded readers. Three, two, and three TNBC lesions were categorized to BI-RADS 3 by three readers, respectively [38]. Even if the combination of ultrasound elastography with Doppler ultrasound is performed, not all TNBC lesions can be identified [38]. In view of the highly invasiveness and poor prognosis of TNBC, more and more imaging methods were used to distinguish TNBC [39–41]. Ultrasound elastography [39], contrast-enhanced ultrasound [40], and analysis of breast ultrasound images by machine learning [41] were used for TNBC detection, and some of the latest work may enhance the image analysis in the US examination [42].

The major limitation of our study was inherent and unchangeable disadvantages of retrospective study. The accuracy of ultrasound examination depends on careful and real-time observation. In the retrospective studies, the inspection process was not restored. Meanwhile, the sample size of the present study was relatively small. In further study, we will enroll more cases in multicenter to validate the findings of the present study. The data of ultrasound elastography and contrast-enhanced ultrasound are very limited in the current study.

## 5. Conclusions

In the retrospective study, the most common TNBC lesion was a hypoechoic mass with an irregular shape and ill-defined margin. In comparison to non-TNBC, the distinctive ultrasonic characteristics of TNBC were summarized as three features: less calcifications, posterior acoustic, and blood flow. Another characteristic of TNBC is that markedly hypoechoic lesions are significantly common. The US shows good predictive value for malignant lesions. Ultrasound can be considered as a useful adjunctive tool for detecting breast cancer. Although TNBC cannot be distinguished from non-TNBC solely on US, some US characteristic provide a more reliable malignant sign. We recommend these characteristics be evaluated in further multicenter, prospective studies for their diagnostic performance for TNBC.

## Figures and Tables

**Table 1:** Characteristics of patients with TNBC versus non-TNBC.

	TNBC patients (*N* = 162) lesion (184)	Non-TNBC patients (*N* = 174) lesion (196)	*P* value
Age (years)	50.58 ± 10.30	49.75 ± 9.12	0.43

Age<40 yrs	24	19	

Size of lesions measured by US			
D1(mm)	23 (15, 31)	21 (15, 33)	0.63
D2 (mm)	16 (11, 23)	15 (11, 21)	0.20
D1/D2<1	25 (13.59%)	29 (14.80%)	

Mass shape			0.67
Irregular	152 (83.06%)	160(81.63%)	
Oval	28(15.30%)	33(16.84%)	
Regular	1 (0.55%)	1(0.51%)	
Indistinct	1 (0.55%)	0	
Flaky lesions	1 (0.55%)	0	
Round	0	2(1.02%)	

Mass margins			0.061
Well-defined	37 (20.22%)	28 (14.29%)	
Angular	28 (15.30%)	31 (15.82%)	
Crab-like	21 (11.48%)	43 (21.94%)	
Indistinct	39 (21.31%)	36 (18.37%)	
Microlobulated/lobulated	35 (19.13%)	42 (21.43%)	
Spiculated	23 (12.57%)	16 (8.16%)	

Echogenicity			<0.001
Hypoechoic lesions	129 (70.11%)	183 (93.37%)	
Mix-hypoechoic lesions	9(4.89%)	3 (1.53%)	
Markedly-hypoechoic lesions	44 (23.91%)	8 (4.08%)	
Anechoic lesions	2 (1.09%)	1 (0.51%)	
Hyperechoic lesions	0	1(0.51%)	

Calcifications			0.03
None	109 (59.24%)	90 (45.91%)	
Microcalcification	64(34.78%)	95 (48.47%)	
Macrocalcification	10(5.43%)	9 (4.59%)	
Microcalcification and macrocalcification	1 (0.54%)	2 (1.02%)	

Posterior features			<0.001
No posterior acoustic feature	136 (73.91%)	128 (65.31%)	
Enhancement	28 (15.22%)	5 (2.55%)	
Attenuating	20 (10.87%)	63 (32.31%)	

The lesion's effect on the surrounding tissue			0.40
No change	159 (86.41%)	167 (85.20%)	
Structural distortion	12 (6.52%)	9 (4.59%)	
Ductal dilatation	1 (0.54%)	2 (1.02%)	
Edema of skin or surrounding tissue	4 (2.17%)	12 (6.12%)	
Changes of cooper ligament	2 (1.09%)	1 (0.51%)	
Others	6 (3.26%)	5 (2.55%)	

Blood flow			<0.001
Avascular	40 (21.74%)	2 (1.02%)	
Strip	110 (59.78%)	147 (75.00%)	
Spot	24 (13.04%)	47 (23.98%)	
Rich strip	10 (5.43%)	0	
Resistance index (TNBC = 39, non -TNBC = 58)	0.78 ± 0.41	0.77 ± 0.07	0.91
Resistance index >0.6	35	58	

Classification of TNM			
Tis/T1/T2/T3/T4	0/54/84/8/15	1/58/93/13/9	
N0/N1/N2/N3	80/53/15/12/1	73/58/25/15/3	
M0/M1	156/5	167/7	

Tumor type			
Invasive ductal	124	154	
Ki67 (%)	60 (40, 75)	35 (20, 50)	<0.001

^*∗*^A lesion was too large to measure. Some data on TNM were missing.

**Table 2:** BI-RADS assessed by the US and mammography.

	US	Mammography
TNBC		(*N* = 122)
0		2
1		1
2		1
3	12	6
4A	28	5
4B	41	41
4C	49	37
5	54	29

Non-TNBC		(*N* = 155)
0		2
1		1
2		4
3	11	3
4A	52	3
4B	29	39
4C	51	35
5	53	68

## Data Availability

The data used to support the results of this study are available from the corresponding author upon request.

## Supplementary Materials

Supplementary Materials We used “triple-negative” and “breast” to search cases in electronic medical record system for eligible patients, respectively. Finally, we enrolled 162 TNBC patients and 174 non-TNBC patients, respectively.

## References

[1] Siegel R. L., Miller K. D., Jemal A. (2020). Cancer statistics, 2020. *CA: A Cancer Journal for Clinicians*.

[2] Breast cancer committee of Chinese Anti-Cancer Association (2019). Guidelines and norms for breast cancer diagnosis and treatment. *China Oncology*.

[3] Chinese Anti-Cancer Association National Clinical Research Center for Cancer (2019). Breast cancer screening guideline for Chinese women. *Chinese Journal of Clinical Oncology*.

[4] (2021). Guidelines for breast cancer diagnosis and treatment (2021 Edition). Chinese Anti-Cancer Association. *China Oncology*.

[5] Shang M. Y., Guo S. (2020). Current status of breast cancer screening in China. *The Practical Journal of Cancer*.

[6] Peairs K. S., Choi Y., Stewart R. W., Sateia H. F. (2017). Screening for breast cancer. *Seminars in Oncology*.

[7] Buchberger W., Geiger-Gritsch S., Knapp R., Gautsch K., Oberaigner W. (2018). Combined screening with mammography and ultrasound in a population-based screening program. *European Journal of Radiology*.

[8] Dent R., Trudeau M., Pritchard K. I. (2007). Triple-negative breast cancer: clinical features and patterns of recurrence. *Clinical Cancer Research*.

[9] Venkitaraman R. (2010). Triple-negative/basal-like breast cancer: clinical, pathologic and molecular features. *Expert Review of Anticancer Therapy*.

[10] Dass S. A., Tan K. L., Selva Rajan R. (2021). Triple negative breast cancer: a review of present and future diagnostic modalities. *Medicina*.

[11] Schettini F., Giuliano M., De Placido S., Arpino G. (2016). Nab-paclitaxel for the treatment of triple-negative breast cancer: rationale, clinical data and future perspectives. *Cancer Treatment Reviews*.

[12] Xing X., Fan Z., Gao Y., Liu Z. (2021). High prevalence of triple-negative breast cancer in southern Shaanxi Province, China. *Cancer Management and Research*.

[13] Carey L., Winer E., Viale G., Cameron D., Gianni L. (2010). Triple-negative breast cancer: disease entity or title of convenience?. *Nature Reviews Clinical Oncology*.

[14] Yang W. T., Dryden M., Broglio K. (2008). Mammographic features of triple receptor-negative primary breast cancers in young premenopausal women. *Breast Cancer Research and Treatment*.

[15] Gao B., Zhang H., Zhang S. D. (2014). Mammographic and clinicopathological features of triple-negative breast cancer. *British Journal of Radiology*.

[16] Du H. Y., Lin B. R., Huang D. P. (2015). Ultrasonographic findings of triple-negative breast cancer. *International Journal of Clinical and Experimental Medicine*.

[17] Li J. W., Zhang K., Shi Z. T. (2018). Triple-negative invasive breast carcinoma: the association between the sonographic appearances with clinicopathological feature. *Scientific Reports*.

[18] Wang H., Zhan W., Chen W., Li Y., Chen X., Shen K. (2020). Sonography with vertical orientation feature predicts worse disease outcome in triple negative breast cancer. *The Breast*.

[19] Kojima Y., Tsunoda H. (2011). Mammography and ultrasound features of triple-negative breast cancer. *Breast Cancer*.

[20] Ko E. S., Lee B. H., Kim H. A., Noh W. C., Kim M. S., Lee S. A. (2010). Triple-negative breast cancer: correlation between imaging and pathological findings. *European Radiology*.

[21] Choi Y. J., Seong M. H., Choi S. H. (2011). Ultrasound and clinicopathological characteristics of triple receptor negative breast cancers. *J Breast Cancer*.

[22] Tian L., Wang L., Qin Y., Cai J. (2020). Systematic review and meta-analysis of the malignant ultrasound features of triple-negative breast cancer. *Journal of Ultrasound in Medicine*.

[23] Dogan B. E., Gonzalez-Angulo A. M., Gilcrease M., Dryden M. J., Yang W. T. (2010). Multimodality imaging of triple receptor-negative tumors with mammography, ultrasound, and MRI. *American Journal of Roentgenology*.

[24] American College of Radiology (2013). ACR practice guideline for communication of diagnostic imaging findings. http://www.acr.org/%7E/media/ACR/Documents/PGTS/guidelines/Comm_Diag_Imaging.pdf.

[25] Wojcinski S., Soliman A. A., Schmidt J., Makowski L., Degenhardt F., Hillemanns P. (2012). Sonographic features of triple-negative and non–triple-negative breast cancer. *Journal of Ultrasound in Medicine*.

[26] Krizmanich-Conniff K. M., Paramagul C., Patterson S. K. (2012). Triple receptor–negative breast cancer: imaging and clinical characteristics. *American Journal of Roentgenology*.

[27] (2020). *Breast Cancer Committee of China Anti-Cancer Association, NCCN Clinical Practice Guidelines in Oncology*.

[28] Moshayedi A. J., Roy A. S., Kolahdooz A., Shuxin Y. (2022). Deep learning application pros and cons over algorithm. *EAI Endorsed Trans AI Robotics*.

[29] Yang Q., Liu H. Y., Liu D., Song Y. Q. (2015). Ultrasonographic features of triple-negative breast cancer: a comparison with other breast cancer subtypes. *Asian Pacific Journal of Cancer Prevention*.

[30] Li Z., Tian J., Wang X. (2016). Differences in multi-modal ultrasound imaging between triple negative and non–triple negative breast cancer. *Ultrasound in Medicine and Biology*.

[31] Wang D., Zhu K., Tian J. (2018). Clinicopathological and ultrasonic features of triple-negative breast cancers: a comparison with hormone receptor–positive/human epidermal growth factor receptor- 2–negative breast cancers. *Ultrasound in Medicine and Biology*.

[32] Zhu K., Wang D., Li Z. (2020). Heterogeneity of triple-negative breast cancer: differences in clinicopathologic and ultrasound features between premenopausal and postmenopausal patients. *Journal of Ultrasound in Medicine*.

[33] Barreau B., Mascarel I. d., Feuga C. (2005). Mammography of ductal carcinoma in situ of the breast: review of 909 cases with radiographic-pathologic correlations. *European Journal of Radiology*.

[34] Sun C. C., Lenoir G., Lynch H., Narod S. A. (1996). In-situ breast cancer and BRCA1. *The Lancet*.

[35] Schrading S., Kuhl C. K. (2008). Mammographic, US, and MR imaging phenotypes of familial breast cancer. *Radiology*.

[36] Lerma E., Peiro G., Ramon T. (2007). Immunohistochemical heterogeneity of breast carcinomas negative for estrogen receptors, progesterone receptors and Her2/neu (basal-like breast carcinomas). *Modern Pathology*.

[37] Stavros A. T., Thickman D., Rapp C. L., Dennis M. A., Parker S. H., Sisney G. A. (1995). Solid breast nodules: use of sonography to distinguish between benign and malignant lesions. *Radiology*.

[38] Yeo S. Y., Kim G. R., Lee S. Y., Moon W. Y. (2018). Comparison ofUltrasound elastography and ColorDoppler ultrasonography for distinguishing small triple-negative breast cancer from fibroadenom. *Journal of Ultrasound in Medicine*.

[39] Wan J., Wu R., Yao M. (2018). Acoustic radiation force impulse elastography in evaluation of triple-negative breast cancer: a preliminary experience. *Clinical Hemorheology and Microcirculation*.

[40] Fang K., Wang L., Huang H. (2020). Construction of nucleolin-targeted lipid nanobubbles and contrast-enhanced ultrasound molecular imaging in triple-negative breast cancer. *Pharmaceutical Research*.

[41] Wu T., Sultan L. R., Tian J., Cary T. W., Sehgal C. M. (2019). Machine learning for diagnostic ultrasound of triple-negative breast cancer. *Breast Cancer Research and Treatment*.

[42] Mahajan S., Abualigah L., Pandit A. K., Altalhi M. (2022). Hybrid Aquila optimizer with arithmetic optimization algorithm for global optimization tasks. *Soft Computing*.

