# Supplementary figures and images for: Calcification, Posterior Acoustic, and Blood Flow: Ultrasonic Characteristics of Triple-Negative Breast Cancer
Source: J Healthc Eng. 2022 Sep 26;2022:9336185. doi: 10.1155/2022/9336185 (PMC9529478; doi:10.1155/2022/9336185)

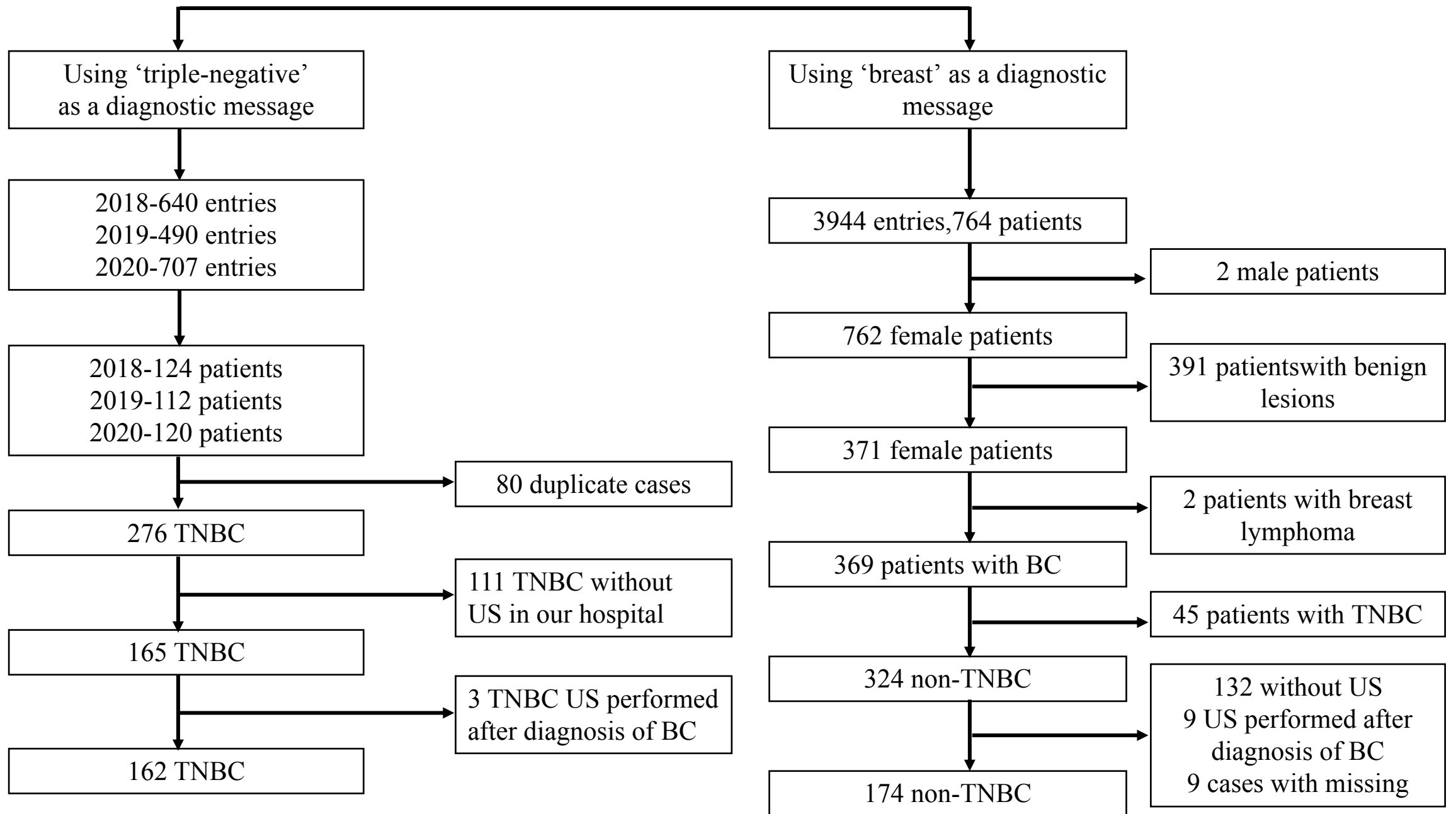

Supplement: Supplementary Materials — We used “triple-negative” and “breast” to search cases in electronic medical record system for eligible patients, respectively. Finally, we enrolled 162 TNBC patients and 174 non-TNBC patients, respectively. [file 9336185.f1.pdf]
